# Who tugs at our heart strings? The effect of avatar images on player generosity in the dictator game

**DOI:** 10.1177/17470218211050359

**Published:** 2021-10-25

**Authors:** Kathryn Buchanan, Jonathan J. Rolison, Isadora Jinga, Jessica Thompson, Riccardo Russo

**Affiliations:** 1 Department of Psychology, University of Essex, Colchester, UK; 2 Department of Brain and Behavioral Sciences, University of Pavia, Pavia, Italy

**Keywords:** Generosity, avatar images, prosocial behaviour

## Abstract

The present research was motivated by a prior study, where several wallets, each containing a photo of either a baby, a puppy, a family, or an elderly couple, were scattered across a city in the United Kingdom. Most of the wallets containing a photo of a baby were returned compared with less than one-third of the wallets containing a photo of an elderly couple. To investigate further, in a series of three studies we examined, using a pseudo online version of the dictator game, possible subtle cues supporting prosocial behaviour by manipulating the type of avatar used by the recipient of the donation made by the “dictator.” Overall, it emerged that participants showed significantly higher levels of generosity towards babies and older people, supporting the notion that perceptions of vulnerability and need drive prosocial behaviour.

## Introduction

In 2009, a team of researchers scattered 240 wallets across the city of Edinburgh in the United Kingdom (Wiseman, 2009). Some of the wallets contained a photo of a baby, others a photo of a puppy, a family, or an elderly couple. In total, 42% of the wallets were returned to local authorities by members of the public. This quasi-naturalistic study raises the intriguing question of whether people’s decisions about whether to return a wallet are influenced by the type of photo it contains. This question is of practical and theoretical importance. On a practical side, charities often depend on public donations and seek methods to promote charitable giving. On a theoretical side, research has emphasised knowledge of the recipient and perceptions of need as key determinants of charitable giving. Is prosocial behaviour also influenced by subtle cues, such as a photo contained in a lost wallet?

The team of researchers discovered that most (88%) of the wallets containing a photo of a baby were returned; fewer wallets containing either a photo of a puppy (53%) or a family (48%) were returned; and less than a third (28%) of those that contained a photo of an elderly couple were returned. Thus, prosocial behaviour seemingly is influenced by subtle cues. Perhaps, as the researchers propose, the photo of a baby elicited a sense of responsibility that encouraged prosocial behaviour (Wiseman, 2009).

Why then is prosocial behaviour influenced by subtle cues as a photo contained in a lost wallet? One possibility is that a photo of an infant baby elicits a sense of responsibility that promotes prosocial behaviour. The infant face possesses infantile features, such as large eyes and prominent cheeks, that have been proposed to elicit parental and caregiving responses from adults (Bornstein et al., 2008; Bowlby, 1969; Lorenz, 1971). In a neuroimaging study, Kringelbach et al. (2008) presented adult participants with photos of faces of unfamiliar adults and infants. Participants exhibited a surge of activity in the medial orbitofrontal cortex on presentation of infant faces, but not adult faces, indicating greater allocation of attention to infant faces, in keeping with the proposal that infant faces trigger special responding from adults. Caria et al. (2012) observed neurological activity in the supplementary motor areas and lateral premotor areas in response to unfamiliar infant faces, suggesting a triggering of preparatory behaviour, which may reflect a readiness to interact with infants. The authors also observed increased activity in the thalamocingulate dopaminergic system and insula in response to unfamiliar infant faces. These brain regions are associated with motivation and reward processing (Strathearn et al., 2008) and processing of emotional stimuli (LeDoux, 2003), which Caria et al. (2012) proposed may jointly underlie the activation of parenting behaviours. Seemingly, this triggering of parental behaviours occurs in adults in response to photos of unfamiliar infants to which adults have no personal responsibility. Moreover, these neurological signatures occur for human infant faces and not for animal infant faces, indicating that the triggering of parental behaviours is species-specific. Thus, a photo of an unfamiliar infant face may trigger parental caregiving behaviours in adults that are automatic due to their evolved neurological underpinnings and which promote prosocial behaviour.

Another possibility is that people perceive some persons or social groups as more vulnerable or in need and that these perceptions of vulnerability or need promote prosocial behaviour. Individuals are more willing to help persons who are identified than they are to help unidentified persons, even when limited (to no) personalising information is provided about the identified persons in need (Small et al., 2007; Small & Loewenstein, 2003). Individuals are also sometimes more willing to help a single person than a group of people (Kogut & Ritov, 2007; Västfjäll et al., 2014; Wiss et al., 2015). These findings suggest that perceived needs of singular identifiable persons elicit emotional reactions that promote charitable behaviour, but that the intensity of emotional reactions diminishes with each additional person, leading to display less charitable behaviour towards groups of people (e.g., Västfjäll et al., 2014). Therefore, we can expect individuals to exhibit the most charitable or prosocial behaviour towards singular identifiable persons to the extent that they are perceived to be in need.

Studies of charitable giving have shown that perception of need is one of the principal motivators of charitable giving (Bekkers & Wiepking, 2011). Committed charity donors often view that “needy people are—and should be—the focus of charitable activity” (Breeze, 2013). Higher perceptions of need are also associated with larger donations to international relief organisations (Cheung & Chan, 2000) and alumni mater (Weerts & Ronca, 2007). For example, potential donors expressed a preference to send aid to countries with higher rates of hunger, malnutrition, and child mortality than to low-income countries in general (Hansen et al., 2014). Perhaps, subtle cues, such as a photo contained in a lost wallet, are sufficient to motivate charitable or prosocial behaviour by eliciting perceptions of need. Adults perceive children, and especially their own children, to be more vulnerable than they truly are (Thomasgard & Metz, 1995). A photo of an infant may then elicit perceptions of need that in turn promote prosocial behaviours. This possibility implies that the images of infants elicit perceptions of vulnerability or need, rather than trigger evolved parental behaviours. Moreover, if images of infants elicit perceptions of vulnerability or need, then similar levels of charitable or prosocial behaviour should be shown for other persons who are perceived to be vulnerable or in need, regardless of their age. The principal aims of current research are to investigate (a) whether subtle cues, such as an image of an infant, elicit charitable or prosocial behaviour and (b) whether in such instances, charitable or prosocial behaviour is driven by the triggering of automatic evolved parental behaviours or perceptions of vulnerability or need that underlie a broad array of charitable and prosocial behaviour. To address the above aims, we will use the dictator game.

**Figure 1.**
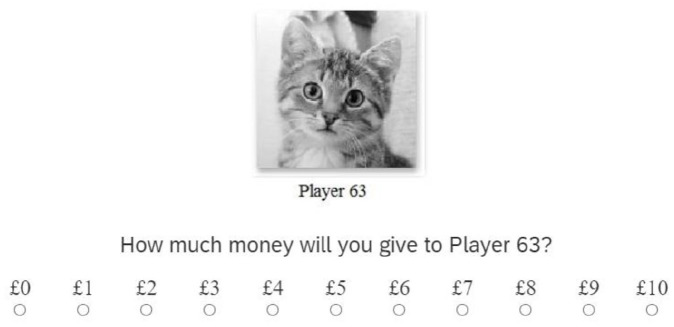
Example of presentation format of the dictator game.

The dictator game offers one instance in which people may act generously even when selfish behaviour is rewarded. Accordingly, in the current research, we use the dictator game as a tool to investigate whether subtle cues can elicit prosocial behaviour. In the dictator game, a single player, the proposer, is asked to decide how much of a fixed sum of money (e.g., US$10) they wish to share with another player (see Figure 1; Engel, 2011; Forsythe et al., 1994; Kahneman et al., 1986). The recipient cannot refuse the share offered to them and can neither return the favour nor punish the proposer. Moreover, typically both players are anonymous and thus selfishness is not associated with any negative outcomes for the proposer. Despite the opportunity to act selfishly, more than half of the participants share some of their endowment, sharing on average 20%–30% (Camerer, 2003; Forsythe et al., 1994). Participants’ generosity may partly reflect motivations to maintain fairness or avoid inequality in social situations and/or personality factors, such as honesty–humility (e.g., Hilbig et al., 2015).

When effort is made to ensure the anonymity of the proposer, such that even the experimenter is unaware of the proposer’s decision, fewer than half of participants share any of their endorsement with the other player (Hoffman et al., 1996).

Even fewer proposers (33%) share with the other players in dictator games when they are offered an “exit” that incurs a small cost to hide the game from the other player, such that they are never told that the game took place (Dana et al., 2006). Generosity in dictator games, then, may result primarily not from concern about another person’s welfare, but from concern about appearing selfish and violating others’ expectations about fairness and equality. Indeed, Haley and Fessler (2005) found that presenting an eye-like image on the computer monitor operated by proposers doubled the likelihood that they would share their endowment, presumably by cueing thoughts about the presence of others. Yet, some prosocial tendencies remain in dictator games even after minimising opportunities for the proposer to present to others an image of themselves as altruist (Gintis et al., 2003; Henrich & Fehr, 2003). Seemingly, people are disposed to certain kinds of prosocial behaviour, such as being altruist (Hilbig et al., 2015).

## Current research

In the current research, we use the dictator game as a tool to investigate whether subtle cues elicit charitable or prosocial behaviour, and whether such behaviour is driven by automatic evolved parental behaviours or perceptions of vulnerability and need.

In Study 1, participants chose among photos of avatars to depict themselves in a dictator game and were asked how much of a fixed amount they would hypothetically share with other players in the game depicted by photos of avatars they had supposedly chosen to represent themselves in the game. Avatars included photos of human infants, nonhuman infants, and older people. If images of human infants trigger automatic parental behaviours, then participants should show greater generosity to other players depicted by a human infant than by another avatar. If elderly and babies command the same level of generosity, then vulnerability is the likely driver of generous behaviour. Finally, avatars of baby animals were used to control for a possible cuteness effect. If cuteness, rather than vulnerability, is the key driver of generous behaviour, then donations to puppies and babies should be comparable and greater than donations received by the elderly. Avatars of doors were included as an inanimate control group.

Study 2 is designed to disentangle parental behaviours from perceptions of vulnerability or need by manipulating apparent vulnerability or need. Vulnerable versions of avatars used in Study 1 are included that depict facial abnormalities (e.g., cleft palate, tumorous growth). In doing so, Study 2 enables us to measure the influence of perceived vulnerability or need on generosity in the dictator game. In Studies 2 and 3, we also include a measure of social desirability to examine the extent to which participant’s generosity is driven by a desire to avoid appearing unselfish and unfair. In Study 3, we probe participants’ strategies by asking them to further explicate the decision-making strategies underlying their behaviour. These studies were approved by the University of Essex Faculty of Science and Engineering Ethics Committee. All participants gave informed consent to take part in the study.

## Study 1: method

### Participants

A total of 168 British citizens (28% males) aged 18 to 77 (*M* = 23.25, *SD* = 7.88) years were recruited on a voluntary basis to complete the online study. The majority of respondents were students (70%). Of these, 16% were psychology majors.

### Procedure

After providing their informed consent, participants were presented with the instructions for the dictator game, which explained that the game would involve deciding how to divide a hypothetical sum of £10 between themselves and another person, randomly selected from a pool of online players. The instructions stated the number of times the game would be played (in this study, 14 times) and emphasised that each player would select an avatar to represent them in the game. Following these instructions, participants were then asked to select one of the seven avatars to represent themselves in the game (a top hat, a sunflower, an adult male, an adult female, a paper clip, a tomato, or a cupcake).

After participants selected their avatar, the dictator game ensued. This involved presenting participants with an avatar labelled with the player number (e.g., “Player 101”). Below it, the question “How much money would you give to Player 101?” appeared and directly underneath this, the participants were able to select a fictitious sum to allocate in £1 increments from £0 to £10 (see Figure 1). To increase participants’ belief that they were playing with other “real players,” after deciding how much to allocate each avatar participants were presented with the following message: “Please wait while we find you another player.” After a pre-specified time interval ranging between 0 and 7 s, participants were presented with the next avatar and were asked how much money (i.e., of £10) they would allocate to them. The average amounts allocated by the participants during the game were used as a measure of their generosity towards each avatar category. Following the game, participants provided their demographic information and were asked what they thought was the purpose of the study.^1^ At the end of the study, participants were thanked for taking part in the study and no debrief was given.

### Materials: details of avatars presented

The stimuli comprised 28 distinct images: 6 per each of the following 4 categories: babies, older people (i.e., above 80 years), baby animals, and doors. Four other inanimate objects were added as filler items to disguise the purpose of the study; these included windows (2 images) and office stationery (2 images). These 28 images were evenly distributed into one of the two picture sets: Picture set A or Picture set B. Participants were randomly allocated to view just one of these picture sets. In both picture sets, the black and white images were sized 6 × 6 cm^2^ and appeared in the same set order.^2^ Where images of humans were used as the images focused on the face of the individuals pictured. Care was taken to avoid selecting celebrities or other public facing figures that may be recognisable to the general public. Only one avatar image was shown per round of the dictator game. Participants could not return to earlier avatars to amend prior responses.

### Analytic strategy

We first performed a frequentist analysis of variance (ANOVA) with appropriate post hoc follow-up tests. Then, we performed the Bayesian equivalent to obtain a Bayes factor (BF) for the overall ANOVA and pairwise comparisons. To calculate the BF, we used JASP in its default settings for the a priori distribution of the parameters (JASP Team, 2017; Wagenmakers et al., 2018). BF can be used as an index to quantify the degree of evidence in favour of either the null or the alternative hypothesis (Hoekstra et al., 2018). BF’s values comprised between 3 and 10 indicate moderate support for the alternative hypothesis (while values larger than 10 provide stronger support), while values comprised between 1/3 and 1/10 indicate moderate support for the null hypothesis (while values smaller than 1/10 provide stronger support). BF values comprised between 1/3 and 3 are inconclusive. With respect to the frequentist ANOVAs reported, we also estimated that, assuming a medium effect size, i.e., *f* = 0.25, the total sample size required to achieve a power of at least 0.8 to detect significant main effects across conditions in each of the three studies was about 45.

## Study 1: results and discussion

On average, participants indicated that they would share £3.69 (*SD* = 1.92) of the £10 endowment offered to them. However, avatar category influenced participants’ generosity. Participants shared the greatest amount with an avatar of an older person (*M* = £4.59; *SD* = 2.63), followed by an avatar of a human baby (*M* = £4.45; *SD* = 2.68), and a baby animal (*M* = £3.76; *SD* = 2.62), and shared the least with inanimate objects (*M* = £2.82; *SD* = 2.07).

The effect of avatar category was confirmed by a significant one-way repeated-measures analysis of variance (ANOVA), *F*(3, 501) = 34.64, mse = 3.17, *p* < .001, partial η^2^ = .17, following Greenhouse–Geisser adjustment, (BF > 100). Pairwise comparisons, corrected according to the Bonferroni procedure to keep the family wise error rate at *p* ⩽ .05, confirmed significant differences between each of the avatar categories (with BFs > 14), except between avatars of an older person and a human baby (BF = 0.11).

Overall, the results of Study 1 provide initial support for the vulnerability hypothesis insofar as avatars depicting human babies and elderly people received the largest amount of money. However, there is little evidence for the explanation put forward that the unique kindchenschema features of a baby face (large head, round face, and big eyes) trigger a deeply rooted evolutionary response that prompts caregiving behaviours. This is because elderly people’s faces do not have these kindchenschema features, yet the generosity shown towards them did not significantly differ from the generosity shown towards human babies. In addition, avatars depicted by baby animals received a significantly smaller share than either avatars of human babies or older people, suggesting that the cuteness factor, may be not as important as the vulnerability factor in inducing generous behaviour. However, please notice that this conclusion rests on the strong untested assumption that baby animals are mostly perceived as cute rather than vulnerable.

## Study 2: method

In Study 1, we found that participants were more inclined to show generosity towards babies and older people than to other avatars. The common denominator between these two categories is vulnerability. However, it also appears that these two categories were the only ones involving human faces, so it is essential to introduce other human categories, not to be considered vulnerable, to further examine whether vulnerability was a viable explanation. Hence, in Study 2, we manipulated vulnerability by including avatars from each of the age categories used. Moreover, to babies and old adults, we added the adult category, and in each age group, half of the avatars appears with and the other half without impairments. Impairments included vulnerabilities that were visible in either structural facial abnormalities (e.g., cleft palate, tumorous outgrowths, Turner syndrome, and Down syndrome), skin conditions (e.g., acne, scarring, and birth mark), or facial expressions (e.g., dysphoria and Parkinson’s disease).

As our avatar categories include vulnerable and non-vulnerable avatars for each of the age categories (i.e., older people, adults, and babies), it is possible that participants will share more with players associated with vulnerable avatars to present a positive social image of themselves. Previous studies have shown generosity in dictator games to be influenced by desire to maintain others’ expectations about fairness and desire not to appear selfish (Dana et al., 2006; Hoffman et al., 1996). Thus, we also measured individual differences in social desirability to assess whether this factor accounts for some of the variability in generosity.

Finally, we also included, but only for the adults’ avatars, subsets of attractive and normal looking people. This to assess, as a secondary aim of the research, whether attractiveness could be a subtle cue to influence performance in the dictator game. However, we do not report the results of avatar attractiveness as this variable, as shown below, did not seem to have any significant impact on performance.

### Participants

A total of 100 respondents (27% males), aged 18 to 67 (*M* = 29.96, *SD* = 9.98) years, completed the study online. The majority of participants (81%) were British citizens. Of the entire sample, 38% classified themselves as students, with 17% reporting that they majored in psychology.

### Measures

#### Generosity

Participants indicated how much of £10 they would give to each “player” by selecting their chosen response on an 11-point scale, ranging from £0 to £10.

#### Social desirability

The revised form of the social desirability scale-17 (SDS-17R; Stöber, 2001) was used to assess social desirability. It contains 16 statements, describing socially desirable but infrequent behaviours (e.g., “I always eat a healthy diet”) and socially undesirable but frequent behaviours (e.g., “I take out my bad moods on others now and then”—reverse scored). Participants indicated whether each statement described them by selecting either true or false. Social desirability scores were summed across the 16 items (*M* = 8.34; *SD* = 2.94).

### Materials: details of avatars

The stimuli comprised 42 distinct images: 6 per each of the following of 7 avatar categories: (1) babies, (2) vulnerable babies, (3) good-looking adults, (4) average-looking adults, (5) vulnerable adults, (6) older people, and (7) vulnerable older adults. These 42 images were evenly distributed into one of the two picture sets: Picture set A or Picture set B. Participants were randomly allocated to view one of these picture sets, in either one of two orders.

In Study 1, participants chose their avatar from a list of “neutral” avatars (e.g., sunflower, cupcake, and tomato) that were not represented in the game. In Studies 2 and 3, participants selected their avatars from a list that included an image from each of the avatar categories they would encounter later on in the game. We did this with the hope that it may have increased participant’s belief in the existence of the “other player” as they would be represented by similar stimuli that they too had a chance to select to represent them.

We conducted a pilot study (*N* = 20; 40% male; aged 19–50 years, *M* = 30.90; *SD* = 10.17) to confirm differences in perceptions of vulnerability according to avatar age and between vulnerable versus non-vulnerable categories. Participants rated the vulnerability of each of the 42 avatar images. Responses were provided on a 10-point scale ranging from 1 (either: *not at all vulnerable*) to 10 (*extremely vulnerable*).

The avatar images of vulnerable (*M* = 8.77; *SD* = 1.50) and non-vulnerable babies (*M* = 8.16; *SD* = 2.10), and vulnerable (*M* = 7.96; *SD* = 0.95) and non-vulnerable older persons (*M* = 6.74; *SD* = 0.92), were rated by participants as more vulnerable than both vulnerable (*M* = 6.77; *SD* = 0.96) and non-vulnerable (*M* = 2.93; *SD* = 1.39) adults. The effect of avatar age on vulnerability ratings was confirmed by a two-way repeated-measures ANOVA that included avatar age (human baby, adult, and older person) and vulnerable versus non-vulnerable avatars categories as factors, *F*(2, 38) = 69.63, *p* < .001, partial η^2^ = .79 (BF > 100). The ANOVA also confirmed that vulnerable avatar categories were rated as significantly more vulnerable than non-vulnerable categories, *F*(1, 19) = 80.45, *p* < .001, partial η^2^ = .79 (BF > 100). In addition, effects of vulnerable versus non-vulnerable avatar categories interacted with effects of avatar age, *F*(2, 38) = 43.52, *p* < .001, partial η^2^ = .70 (BF > 100). Inspecting the mean image ratings, the interaction was driven by particularly large differences in vulnerability ratings between vulnerable and non-vulnerable images of adults.

To ascertain the face validity of the adults’ stimuli in terms of attractiveness, the same sample of 20 people also assessed the adults’ avatars subsets in terms of attractiveness. Responses were provided on a 10-point scale ranging from 1 (*not at all attractive*) to 10 (*extremely attractive*). A paired-samples *t*-test was conducted on the collected scores. This revealed that the images selected to represent attractive adults were indeed perceived as significantly more attractive than the images selected to represent regular adults, respectively, *M* = 8.35 versus *M* = 5.99, *t*(19) = 12.65, *p* < .001 (BF > 100). As anticipated, despite a large and statistically significant difference in attractiveness between average-looking and good-looking young adults, there was only a minimal difference in the money allocated to these two categories, £3.56 versus £3.49, respectively (BF = 0.135) in the dictator game. This ancillary comparison is reported here rather than in the results section to simplify the exposition and analyses of the obtained results. Overall, attractiveness plays a limited (to no) role in the findings obtained in the dictator game, thus we collapsed the outcomes for good-looking and average-looking adult in the adult category (consisting thus of 12 images). Therefore, in Study 2, we had avatars of (a) vulnerable versus (b) non-vulnerable people, and within each vulnerability condition, we had avatars of baby, adults, and old adults.

### Procedure

The procedure was identical to Study 1 with the addition, after the dictator game task, to answer the Social Desirability questionnaire. At the end of the study, participants were thanked for taking part in the study, debriefed on the purpose of the study, and were told that they were not playing against actual live people.

## Study 2: results

Table 1 displays the descriptive statistics for player generosity towards each of the avatar categories. Participants shared more with avatars of vulnerable (*M* = 5.90; *SD* = 2.96) and non-vulnerable babies (*M* = 5.09; *SD* = 2.66) and vulnerable (*M* = 5.25; *SD* = 2.83) and non-vulnerable older persons (*M* = 5.22; *SD* = 2.38) than they shared with vulnerable (*M* = 5.07; *SD* = 2.68) and non-vulnerable adults (*M* = 3.53; *SD* = 2.19).

**Table 1.** Monetary allocations to “other players” in the dictator game in Studies 1 to 3.

Avatar category	Study 1		Study 2		Study 3	
	*M* (£)	*SD*	*M* (£)	*SD*	*M* (US$)	*SD*
Babies (regular)	4.45	2.68	5.09	2.66	4.19	2.80
Older people (regular)	4.59	2.63	5.22	2.38	4.10	2.60
Adults (regular)	–	–	3.56	2.21	3.37	2.38
Inanimate objects	2.82	2.07	–	–	–	–
Baby animals	3.76	2.62	–	–	–	–
Attractive adults	–	–	3.49	2.31	3.43	2.51
Vulnerable babies	–	–	5.90	2.96	4.59	3.05
Vulnerable older people	–	–	5.25	2.83	4.10	2.75
Vulnerable adults	–	–	5.07	2.68	3.96	2.68

*SD*: standard deviation.

Study 1: *N* = 168; Study 2: *N* = 100; Study 3: *N* = 177.

A 3 × 2 repeated-measures ANOVA was conducted on participants’ monetary allocations to examine the effect of avatar age (babies, adults, and older adults) and avatar vulnerability (normal, vulnerable) on monetary allocation. The analysis confirmed a significant effect of avatar age, *F*(2, 198) = 28.70, *p* < .001, partial η^2^ = .22 (BF > 100), on monetary allocation. Both babies and older adults elicited significantly higher levels of generosity than adults (respectively, £5.49 and £5.24 versus £4.30, both *t*s > 6.25, both *p*s < .001 [BFs > 100]). There was no significant difference between older adults and babies, *t*(99) = 1.50, *p* > .10, (BF = 0.33).

The analysis also confirmed a significant effect of vulnerability category, *F*(1, 99) = 16.98, *p* < .001, partial η^2^ = .15 (BF > 100). Avatar age and vulnerability category significantly interacted, *F*(2,198) = 17.00, *p* < .001, partial η^2^ = .15 (BF > 100). Paired-sample *t*-tests revealed that participants shared significantly more with players associated with a vulnerable than a non-vulnerable baby, *t*(99) = 3.10, *p* < .01 (BF = 9.5), and with a vulnerable than a non-vulnerable adult, *t*(99) = 5.45, *p* < .001 (BF > 100). In contrast, they did not share significantly more money with players associated with a vulnerable than a non-vulnerable older adult, *t*(99) = .16, *p* > .05 (BF = 0.11). Each of the correlations between individual differences in social desirability and generosity shown towards each of the avatar categories were not significant (Pearson’s *r* ranged: −.01 to .18, see Table 2), with the exception of attractive adults (*r* = .21, *p* < .05).

**Table 2.** Correlations between the social desirability scale and monetary allocation to other players in the dictator game.

Avatar category	Study 2	Study 3
Babies (regular)	−.03	.04
Older people (regular)	.11	.06
Adults (regular)	.18	−.01
Attractive adults	.21*	−.00
Vulnerable babies	.11	.10
Vulnerable older people	.08	.08
Vulnerable adults	.05	.10

Study 2: *N* = 100; Study 3: *N* = 177. *p < .05.

## Study 2: discussion

The results obtained in Study 2 further support the primary role of vulnerability in supporting generosity in this version of the dictator game. Avatars of people rated high in vulnerability (i.e., babies, and older adults—and those with disfigurements) tended to receive more money than those who received lower vulnerability ratings. The non-significant correlations between social desirability and monetary allocations failed to provide support for the notion that participant’s behaviour was driven by a desire to avoid appearing unselfish and unfair.

## Study 3: method

### Participants

We recruited citizens of the United States using a convenience sampling method through Amazon’s Mechanical Turk. All participants received a nominal payment for completing the study, regardless of their choices in the dictator game. A total of 177 participants (79 males, 98 females) aged from 19 to 37 (*M* = 36.44, *SD* = 9.90) years completed the online study.

### Procedure

The procedure was identical to Study 2, but included additional questions to elicit further information about the reasoning behind participant’s allocations in the dictator game. At the end of the study, participants were thanked for taking part in the study and no debrief was given.

### Measures

As in Study 2, we assessed participants’ reported generosity towards each avatar and social desirability. The social desirability scale had a mean of 8.25 (*SD* = 4.09) and a Cronbach alpha of .83. In addition, we asked participants about their perceptions of the dictator game. These questions appeared in the order that they are listed below.

#### Dictator game strategy

Participants were asked to describe how they decided to allocate money to each player during the dictator game by providing a written response to the following question: “During the dictator game you were asked to allocate a sum of US$10 between you and another player. On what basis did you decide how much money to give to each player?”

#### Influence of avatar characteristics on generosity

Participants were asked to indicate the extent to which they felt certain characteristics of the avatars had influenced their monetary allocations in the dictator game by responding to the following items: “I gave more money when I felt the avatars looked . . .”: “cute”; “vulnerable”; “happy”; “sad”; “sexually attractive”; “average looking”; “in need of extra help”; “attractive”; “similar to me”; and “unfortunate.” Responses were given for each of the 10 characteristics on a 7-point scale ranging from 1 (*strongly disagree*) to 7 (*strongly agree*). As the items “vulnerable,” “unfortunate,” and “in need of extra help” are conceptually similar, a single “vulnerability” score was calculated as the mean of these three items. The reliability was excellent (Cronbach α = .96).

#### Perceptions of the dictator game

Participants indicated to what extent they agreed/disagreed with the following three statements: (1) “While playing the dictator game, I consciously thought about the fact that each player had chosen the avatar to represent them”; (2) “It felt like I was deciding how much money to give the person in the picture (i.e., the avatar) rather than deciding how much money to give to the other player”; and (3) “I played the dictator game with other online players.” Responses to each of the statements were given using a 7-point scale ranging from 1 (*strongly disagree*) to 7 (*strongly agree*).

## Study 3: results

### Decisions in the dictator game

Table 1 displays the descriptive statistics for player generosity towards each of the avatar categories. As in Study 2, we found no significant difference in the amount of money allocated to attractive versus average-looking adults, 3.43 versus 3.37; *F*(1, 99) = 0.41, NS (BF = 0.107), so we computed a mean of these two categories that represented adults.

A 3 × 2 ANOVA was conducted on participants’ monetary allocations to examine the effect of avatar age (babies, adults, and older adults) and avatar vulnerability (normal, vulnerable) on generosity. The results showed a significant main effect of avatar age category, *F*(2, 352) = 28.77, *p* < .001, partial η^2^ = .15 (BF > 100), and vulnerability, *F*(1, 176) = 8.45, *p* < .001, partial η^2^ = .05 (BF > 100). An interaction between age and vulnerability was also significant, *F*(2, 352) = 8.94, *p* < .001, partial η^2^ = .05 (BF = 1.85). In general, the results replicate the findings obtained in Studies 1 and 2, as older people and babies elicited significantly higher levels of generosity than adults, respectively, US$4.39 and US$4.10 versus US$3.67, both *t*s > 4.85, *p*s < .001 (BFs > 100); unlike Studies 1 and 2, in this instance, babies were found to provoke significantly higher levels of generosity than older people, *t*(177) = 3.36, *p* < .001 (BF = 18.3). Interestingly, if the BFs obtained in the three studies regarding the comparisons of donations with older adults and baby avatars are multiplied, we obtain a BF of 0.66 which is inconclusive with respect to a possible age effect on generosity.

As in Study 2, vulnerable babies and vulnerable adults elicited higher levels of generosity than their non-vulnerable counterparts, vulnerable babies = US$4.58 versus regular babies = US$4.19, *t*(176) = 2.85, *p* < .005 (BF = 4.18); vulnerable adults = US$3.96 versus regular adults = US$3.37, *t*(176) = 3.66, *p* < .001 (BF = 16.14), whereas vulnerable (US$4.10) and normal (US$4.10) avatars depicting older people did not differ significantly in the monetary amount allocated to them, *t*(176) = −.02, BF = 0.084.

Social desirability did not appear to play a critical role in the generosity shown towards the avatar categories (Pearson’s *r* ranged: −.01 to .10).

### Participants’ perceptions of how avatar characteristics influenced their dictator game decisions

When explicitly asked to indicate the extent to which they felt certain avatar characteristics had influenced their monetary allocations, participants were reluctant to agree that any of the characteristics had strongly influenced their decision-making. Table 3 provides the mean ratings. The highest mean rating was for the “vulnerable” characteristic, which participants indicated they “neither agreed nor disagreed” (i.e., mean rating close to 4) had influenced their monetary allocation to other players. However, despite this, the relative differences in ratings for the characteristics are in line with the pattern of results obtained during the dictator game. In the dictator game, attractive avatars elicited the lowest levels of generosity, and similarly, participants rated attractiveness as one of the characteristics least likely to elicit generosity (Table 2). Participants’ monetary allocations were also in line with their ratings that they tended to allocate more money to players they perceived to be vulnerable. Table 2 also provides the correlations between participants’ ratings of the avatar characteristics and the amount of money allocated to each avatar category in the dictator game. This analysis shows that there were significant correlations between participants’ ratings of the avatar characteristics that they judged to elicit the highest levels of generosity and the actual levels of generosity shown in the dictator game. For instance, the vulnerability characteristic was rated as the strongest influence on their decisions to allocate money and was strongly associated with the amount of money given to vulnerable avatars and normal avatars that depicted babies and older people.

### Strategies used during the dictator game: qualitative findings

We used participants’ written responses to the question: “On what basis did you decide how much money to give each player?” to conduct a thematic analysis, as described by Braun and Clarke (2006). We employed an inductive approach consistent with an essentialist method whereby the themes identified were strongly linked to the data. That is, the themes were largely identified at the semantic level.

Table 4 provides the results of the thematic analysis. Participants’ most frequent response was that they allocated money according to the perceived need of the other player. While many responses did not specify which players were perceived to be most in need, some participants did specify that they allocated more money to babies and the elderly, avatars depicting individuals with disfigurements, and ones depicting individuals who looked distressed. Other common strategies included equality (i.e., dividing money equally) or mostly equality (i.e., dividing money evenly, but making exceptions for those deemed most in need), selfishness (i.e., keeping all the money), and attractiveness. Of the responses categorised under attractiveness, approximately half indicated that attractiveness increased generosity while the remaining half reported the opposite, that they allocated less money to those perceived as attractive because they felt them to be less deserving. Less frequently reported strategies included random allocation (i.e., allocating the funds whimsically), listening to gut instinct, reactionary responses (i.e., giving less to players if they perceived that the avatar had been selected to manipulate them into higher levels of generosity), and similarity (i.e., giving money to players who had selected the same category of avatar).

**Table 3.** Ratings of the avatar characteristics and associations with the amount of money allocated to the avatar categories in the dictator game: Study 3.

Study	Avatar characteristic	*M*	*SD*	Pearson’s *r* correlations between avatar characteristic ratings and money allocated to each avatar category							
				Overall generosity	Vulnerable babies	Vulnerable older people	Vulnerable adults	Babies	Older people	Adults	Attractive adults
3	Vulnerable	4.01	2.21	.27**	.45**	.35**	.36**	.27*	.31**	−.05	−.04
	Cute	3.36	2.13	.14	.07	−.001	−.009	.31**	.13	.12	.22**
	Sad	3.35	2.04	.22**	.37**	.32**	.32**	.22**	.27*	−.09	−.10
	Happy	2.90	1.85	.13	.03	.05	.00	.21**	.13	.20**	.22**
	Average looking	2.89	1.72	.11	.05	.07	.02	.17*	.10	.15*	.17*
	Attractive	2.74	1.90	.06	−.09	−.05	−.09	.11	.05	20**	.28*
	Similar to me	2.73	1.75	.11	.01	.05	.00	.16*	.12	.17*	.18*

*SD*: standard deviation.

* *p* < .05; **p < .01; Study 3: *N* = 177; Participants used a 7-point scale ranging from 1 (*strongly disagree*) to 7 (*strongly agree*) to indicate the extent to which they gave more money when they felt the avatars had each of the listed characteristics.

**Table 4.** Thematic analysis of participants explanations of who they decided to allocate funds to during the dictator game in Study 3.

Theme	Illustrative quotes
Perceived need (44%)	ID 153: “I based it on their appearance and how much I thought they would need it” ID 89: “I was looking at who would really need the money”
Equality or mostly equality (35%, respectively 28% and 6%)	ID 15: “Fairness. US$5 for me and US$5 for the other player” ID 40: “I just tried to treat everyone fairly and split the money evenly” ID 158: “I started out splitting it evenly but as I went along I felt compelled to give more to those that appeared to struggle in some way” ID 14: “I usually split it even, we each got five. But for the babies I gave them six. It just felt right”
Attractiveness (11%)	ID 12: “How attractive or how much I liked a person” ID 162: “. . . If they were pretty I gave them less, because pretty people already get more than they deserve”
Selfishness (10%)	ID 1: “I wanted to keep all of the money” ID 105: “I decided not to give any money to anyone and keep it for myself”
Random allocation (5%)	ID 126: “I chose randomly” ID 141: “I just picked a random amount”
Gut instinct (3%)	ID 9: “I wouldn’t say I had any concrete rules, just went with my gut feeling” ID 80: I’m not sure, just intuition”
Reactionary (2%)	ID 76: “I tended to give a little less to those who chose an avatar that seemed to be manipulative to me like a super cute baby, very attractive adult, or disabled or sick person and perhaps a little more to those who chose a more ordinary person.” ID 132: “I feel like showing me your deformity or stressed out old pic is begging for more money so I gave those players less”
Similar to me (1%)	ID 47: “I decided to give them money if they were similar to me based upon if I’d choose that avatar or not”

Percentages provide an indication of the frequency of each theme within the data but will not add up to 100 as participants’ responses sometimes contained multiple themes.

### Perceptions of the dictator game

While across all three studies, efforts were made to disguise the fact that participants were not playing the dictator game against another real online player, it was unclear to what extent participants bought into this belief. When participants were asked directly following the dictator game whether they believed they had been playing against another online player, 47% said that they believed they were, 9% reported that they were unsure, and 45% did not believe that they were. The extent to which participants believed the other player was real did not significantly correlate with the generosity they showed towards any of the avatar categories. This suggest two things: (a) that trying to give participants a mistaken impression about the possible involvements of other players in the dictator game is not very effective in making people believe a receiver actually exist; and (b) more importantly, the above results suggest that either believing or not that a receiver exists does not affect participants’ propensity to donate part of the fictitious money they play within the game. By giving participants a relatively innocuous mistaken impression on the nature of the receiver, we did find the opposite of Frohlich et al. (2001) where it appears that players in a dictator game who suspect that they were not paired with real people give less. Our finding suggests that this is not the case.

When asked whether they consciously thought about the fact that each player had chosen their avatar, 49% said they did, 39% said they did not, and 12% reported not being sure. Similar divisiveness emerged when participants were asked to indicate whether it felt like they were deciding how much money to give to the avatar, with 38% agreeing with the statement, 30% disagreeing, and 37% not being sure either way. These frequencies are in contrast to the qualitative findings as only the reactionary theme indicated that participants might be thinking about the player behind the avatar, while all the other themes suggested it was as though participants felt they were deciding how much money to give the person in the picture.

Correlational analyses confirmed that overall neither thinking consciously about the fact that each player had chosen their avatar nor feeling as though they were allocating money to the person in the picture influenced player generosity in the dictator game. In fact, the only significant correlation was between the generosity shown towards vulnerable babies and the perception that money was being allocated to the person in the picture (Pearson’s *r* = .22, *p* < .01).

### Study 3: discussion

Study 3 replicated our earlier findings as far as participants reported that they would share more of their endowment with players depicted by avatars of human babies and older adults than with others and that they would share more with vulnerable groups than with non-vulnerable groups. Participants also rated vulnerability as the most important characteristic of the avatars in determining their generosity. When describing their reasoning strategy, many participants explained that they were influenced by their perceptions of the other player’s needs in deciding how much to share. Generosity was unaffected by the degree to which participants said they reflected on the player behind the avatar and even whether they believed that there was a real player behind the avatar.

## General discussion

Is the prosocial behaviour shown towards others influenced by subtle cues, such as a photo contained in a lost wallet or an avatar used to represent a person in online environments? In three studies, we asked participants how much (if any) of a monetary endowment they would share with another player associated with various avatars, including an older person, a human baby, a baby animal, an inanimate object (Study 1), a vulnerable baby (with a visible abnormality), a vulnerable older person, and a vulnerable adult (Studies 2 and 3). In doing so, we investigated whether generosity was driven by automated evolved parental behaviours or by perceptions of vulnerability and need. We reasoned that if evolved parental behaviours explain generosity then human infants should receive the highest share of the endowment, whereas if perceptions of vulnerability and need determine generosity then human infants should receive similar levels of endowment to other avatars deemed to be vulnerable, regardless of their age. Across all studies, we found consistent support for the notion that generosity in the dictator game can be influenced by a subtle cue—the avatar of another player.

All of three studies provided considerable support for the notion that perceptions of need and vulnerability determined generosity. Specifically, support for the vulnerability hypothesis is evident in the following three points. First, the amount allocated to babies did not significantly differ from the amount allocated to older adults in Studies 1 and 2. This is in line with the expectation that if perceptions of vulnerability and need determine generosity (Bekkers & Wiepking, 2011; Breeze, 2013) then human infants should receive similar levels of endowment to other avatars deemed to be vulnerable, regardless of their age. Second, avatars with an explicit vulnerability (i.e., a visible abnormality) received higher levels of generosity (Studies 2 and 3) and were rated as significantly more vulnerable than their non-vulnerable counterparts (Study 2). Moreover, participant’s monetary allocations towards these avatar types were not correlated with a measure of social desirability (Stöber, 2001), indicating that experimenter demand does not appear to account for such findings. Third, perceived need emerged as a dominant theme in participants’ explanations of how they decided to allocate funds (Study 3). Moreover, our data are relatively agnostic on the notion that generosity is influenced by automated and evolved parental behaviours elicited by the unique kindchenschema of an infant’s face (Bornstein et al., 2008; Bowlby, 1969; Lorenz, 1971). Only Study 3 showed that babies elicited significantly higher levels of generosity than older adults, but this difference did not emerge when the results from the three studies were considered together.

Finally, by giving participants a relatively innocuous mistaken impression on the nature of the receiver, our results interestingly suggested that, unlike previous findings where players in a dictator game who suspect that they were not paired with real people give less (e.g., Frohlich et al., 2001), in our study either believing or not that a receiver exists does not affect participants propensity to donate part of the fictitious money they play with in the game.

With respect to the above issue of giving wrong impressions about the realness of the other player, as noted by one of the reviewers of this article, this approach may poison a subject pool in the long run and should not be used.

### Limitations and future directions

While we did not find any significant correlations between social desirability and participants decisions in the dictator games in Studies 2 and 3, it is possible that the social desirability measure we used captured a broader desire to convey a good impression to others rather than assessing the more specific motive of appearing fair both to oneself and to others. As such, future research should further investigate this issue, and could employ additional operationalisations of vulnerability (i.e., beyond disfigurement), to increase the generalisability of the results. In the current studies, participants made decisions about hypothetical sums of money. In direct comparisons of “real” and “hypothetical” conditions, participants’ decisions on the dictator game and similar games typically appear to be similar regardless of whether their decisions have real or hypothetical consequences (e.g., Ben-Ner et al., 2008; Locey et al., 2011, see also Engel, 2011). Moreover, our interest was in how avatar characteristics (e.g., vulnerability) influence charitable giving. These within-subjects factors should presumably be even less affected by whether decisions across avatars are hypothetical or real. However, future research could seek to explore whether the hypothetical versus real consequences of participants’ decisions moderate the effects of avatar characteristics on charitable giving.

A potential interesting aspect raised by one of the reviewers refers to the potential endogeneity of the recipient’s avatar choice. Certain faces elicited larger donation amounts. Could it be the avatar chosen by the recipient conveys something about them? If so, it may be interesting in future studies to assess whether something about vulnerability automatically activates a prosocial urge or whether something good is inferred about the person who chooses the avatar.

In summary, the contributions of our study are twofold. First, we demonstrate unequivocally that subtle cues, such as images, can influence participants’ generosity. These effects occurred, even though some of our participants seemed aware that avatars did not depict the player behind them (e.g., in Study 3, 49% of participants reported thinking consciously about the fact that each player had chosen their avatar). It is also possible that participants used the avatar choice to infer something about the player that selected it, thus increasing the identifiability of the recipient and thereby promoting charitable behaviour (Small et al., 2007; Small & Loewenstein, 2003). It is also possible that some participants did not think about each monetary allocation in such depth, but rather responded to the emotional reaction elicited by the images. Either way, it seems to us that there may be a lesson in here about being weary in online environments (e.g., social media, dating sites, and learning forums): images appear to affect the way we interact with others and people should guard against such biases that can be used to manipulate people’s behaviour in subtle ways. Moreover, while images involving vulnerability may elicit *pledges* to act generously, it is worth noticing that the largest donations were always awarded to images depicting vulnerable babies. Such findings have real-world implications for the types of images charities could use in advertising to secure donations.

## Notes

1. Approximately 22% of participants guessed the correct purpose of the study (e.g., ID 108: “To assess how selfish or selfless we are depending on the avatar of the player”; ID 24: “To see how generous you’ll be to a stranger, or if the amount given will depend on the avatar”). Excluding these participants from our analyses did not alter our results. Consequently, we report our findings including all participants, regardless of whether they guessed the purpose of the study or not.

2. The order was as follows: baby, door, older adult, window, puppy, older adult, door, kitten, baby, stapler, chick, older female, door, and baby.
